# DNA multigene characterization of *Fasciola hepatica* and *Lymnaea neotropica* and its fascioliasis transmission capacity in Uruguay, with historical correlation, human report review and infection risk analysis

**DOI:** 10.1371/journal.pntd.0005352

**Published:** 2017-02-03

**Authors:** María Dolores Bargues, Valeria Gayo, Jaime Sanchis, Patricio Artigas, Messaoud Khoubbane, Soledad Birriel, Santiago Mas-Coma

**Affiliations:** 1 Departamento de Parasitología, Facultad de Farmacia, Universidad de Valencia, Valencia, Spain; 2 Departamento de Parasitología, División de Laboratorios Veterinarios (DILAVE), "Miguel C. Rubino", Ministerio de Ganadería, Agricultura y Pesca (MGAP), Montevideo, Uruguay; 3 Departamento de Parasitologia, Universidad de la República (Regional Norte), Salto, Uruguay; 4 Facultad de Veterinaria, Universidad de la República Oriental del Uruguay, Montevideo, Uruguay; Queen's University Belfast, IRELAND

## Abstract

**Background:**

Fascioliasis is a pathogenic disease transmitted by lymnaeid snails and recently emerging in humans, in part due to effects of climate changes, anthropogenic environment modifications, import/export and movements of livestock. South America is the continent presenting more human fascioliasis hyperendemic areas and the highest prevalences and intensities known. These scenarios appear mainly linked to altitude areas in Andean countries, whereas lowland areas of non-Andean countries, such as Uruguay, only show sporadic human cases or outbreaks. A study including DNA marker sequencing of fasciolids and lymnaeids, an experimental study of the life cycle in Uruguay, and a review of human fascioliasis in Uruguay, are performed.

**Methodology/Principal findings:**

The characterization of *Fasciola hepatica* from cattle and horses of Uruguay included the complete sequences of the ribosomal DNA ITS-2 and ITS-1 and mitochondrial DNA *cox*1 and *nad*1. ITS-2, ITS-1, partial *cox*1 and rDNA 16S gene of mtDNA were used for lymnaeids. Results indicated that vectors belong to *Lymnaea neotropica* instead of to *Lymnaea viator*, as always reported from Uruguay. The life cycle and transmission features of *F*. *hepatica* by *L*. *neotropica* of Uruguay were studied under standardized experimental conditions to enable a comparison with the transmission capacity of *F*. *hepatica* by *Galba truncatula* at very high altitude in Bolivia. On this baseline, we reviewed the 95 human fascioliasis cases reported in Uruguay and analyzed the risk of human infection in front of future climate change estimations.

**Conclusions/Significance:**

The correlation of fasciolid and lymnaeid haplotypes with historical data on the introduction and spread of livestock into Uruguay allowed to understand the molecular diversity detected. Although Uruguayan *L*. *neotropica* is a highly efficient vector, its transmission capacity is markedly lower than that of Bolivian *G*. *truncatula*. This allows to understand the transmission and epidemiological differences between Andean highlands and non-Andean lowlands in South America. Despite rainfall increase predictions for Uruguay, nothing suggests a trend towards a worrying human infection scenario as in Andean areas.

## Author summary

Fascioliasis is a highly pathogenic zoonotic disease emerging in recent decades, in part due to the effects of climate and global changes. South America is the continent presenting more numerous human fascioliasis endemic areas and the highest *Fasciola hepatica* infection prevalences and intensities known in humans. These serious public health scenarios appear mainly linked to altitude areas in Andean countries, whereas lowland areas of non-Andean countries, such as Uruguay, only show sporadic human cases or outbreaks. To understand this difference, we characterized *F*. *hepatica* from cattle and horses and lymnaeids of Uruguay by sequencing of ribosomal DNA ITS-2 and ITS-1 spacers and mitochondrial DNA *cox*1, *nad*1 and 16S genes. Results indicate that vectors belong to *Lymnaea neotropica* instead of to *Lymnaea viator*, as always reported from Uruguay. Our correlation of fasciolid and lymnaeid haplotypes with historical data on the introduction and spread of livestock species into Uruguay allow to understand the molecular diversity detected. We study the life cycle and transmission features of *F*. *hepatica* by *L*. *neotropica* of Uruguay under standardized experimental conditions to enable a comparison with the transmission capacity of *F*. *hepatica* by *Galba truncatula* at very high altitude in Bolivia. Results demonstrate that although *L*. *neotropica* is a highly efficient vector in the lowlands, its transmission capacity is markedly lower than that of *G*. *truncatula* in the highlands. On this baseline, we review the human fascioliasis cases reported in Uruguay and analyze the present and future risk of human infection in front of future climate change estimations.

## Introduction

The impact of climate change and global change is putting trematodiases in one of the main focuses of infectious disease actuality [1–4]. Among the food-borne trematodiases emphasized in the recent WHO Roadmap for neglected tropical diseases 2020 [5], fascioliasis depicts a specific place due to its worldwide distribution, emergence, and estimated 17 million people infected throughout [6]. The climate change impact on fascioliasis is linked to the high dependence of both fasciolid larval stages and their freshwater lymnaeid snail vectors on climatic and environmental characteristics [2,7,8]. Additionally, fascioliasis emergence appears also related to global change aspects, such as import/export and management of livestock [6], anthropogenic modifications of the environment [9], travelling [10] and changing human diet traditions [11].

Fascioliasis morbidity in humans has been highlighted by the World Health Organization [12]. The acute and long-term chronic phases of this disease show high pathogenicity and immunosuppressive capacity [13–17]. Aspects adding concern are the clinical complexity and severity of symptoms and syndromes, important sequelae and even death [18], pronounced diagnosis difficulties [19] and treatment problems [20].

South America stands out due to the high human prevalences and intensities reported in Andean countries as Bolivia [21–25], Peru [26–28] and Chile [29,30], and the cases from Ecuador [31], Colombia [32], and Venezuela [33]. However, in the non-Andean, lowland countries, human reports only concern sporadic and isolated cases, such as in Brazil [34] and Uruguay [35].

**Fig 1:**
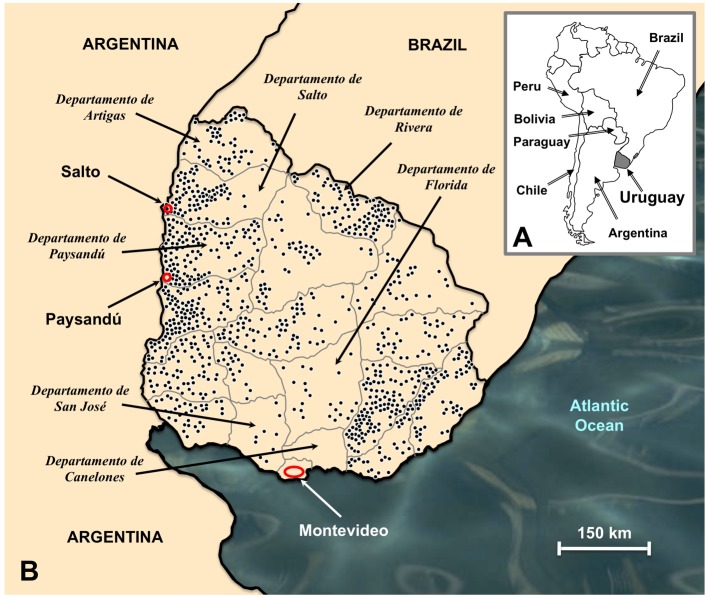
Localities of Uruguay including an overview of the geographical distribution of fascioliasis infection risk. **A.** Location of Uruguay in South America. **B.** Map of Uruguay showing localities and departments of Montevideo, Salto, Paysandú and Canelones where the fasciolid flukes and the lymnaeid snails were collected. Other departments highlighted are those where most of the human fascioliasis cases have been reported. Dots correspond to farms where more than 20% of the cattle was known to be infected by *Fasciola hepatica* according to data from the 1972–1973 period (modified from [37]).

Uruguay has a wide farming and agricultural sector, with 70% of the export trade corresponding to livestock products and subproducts. *Fasciola hepatica*, locally known as “saguaypé” [36], is distributed throughout the large flat lowlands of the whole country (Fig 1). Cattle and sheep are the most affected, which is related to mixed grazing [37], with high prevalences [37–41] and great impact and economic losses [42]. Horses, sharing the same pastures with cattle and sheep, are the third affected species [43,44]. The liver fluke also infects wild rodents, including the capybara *Hydrochoerus hydrochaeris* (Caviidae) [45] and the nutria or river rat *Myocastor coypus* (Myocastoridae) [46]. Fasciolid eggs have also been found in the wild Pampas deer *Ozotoceros bezoarticus* (Cervidae) [47].

There is a direct effect of altitude on fascioliasis transmission [28]. High altitude lymnaeid vectors produce a higher number of metacercariae throughout a longer cercarial shedding period [48]. This higher transmission capacity is related to the longer life span and post-infection survival of the vector in such altitudes [48,49]. The high fascioliasis transmission in human fascioliasis endemic areas in Andean highlands appears linked to the *Galba*/*Fossaria* group species *Galba truncatula*, a very efficient vector of European origin introduced 500 years ago with the livestock transported by the Spanish "conquistadores" [6,48].

Recent reports indicate that other *Galba*/*Fossaria* species may also be involved in human fascioliasis endemic areas, such as *Lymnaea neotropica* in Peruvian highlands [50], and in extreme aridity-dryness habitats in Argentina [51] together with *L*. *viator* (= *L*. *viatrix*—for nomenclature see [30]). The later species is also involved in other places of Argentina [52], whereas it was confused with *G*. *truncatula* in Chile [30] due to the difficulties in phenotypically differentiating between species of *Galba*/*Fossaria* [53].

In Uruguay, only two lymnaeid species have been reported [54: *L*. *viator* [55,56] and *Pseudosuccinea columella* [57]. *Pseudosuccinea columella* has been detected in 14 of the 19 departments of the country [36], even naturally infected [58]. Although known as an important lymnaeid for the disease transmission to livestock [32], veterinary responsibles have assigned only secondary importance to this species in Uruguay. This is because of its sporadic natural infection linked to its different ecology and pronouncedly lower cercarial shedding when compared to *L*. *viator* (a maximum of 10 cercariae/snail in *P*. *columella*; between 100 and 200 cercariae/snail in *L*. *viator*). Therefore, all efforts have always been conducted to ascertain the epidemiological role of *L*. *viator*, both in nature and experimentally in the laboratory [36,59–66].

Three recent findings suggest the need to review the situation in Uruguay: (i) the involvement of *Galba*/*Fossaria* species as *L*. *viator* and *L*. *neotropica* in human fascioliasis endemic areas [50,51]; (ii) their experimentally verified high transmission capacity [67]; and (iii) the potential impact of climate change on fascioliasis and *Galba*/*Fossaria* species [1,2,9], given the predictions on the climate change impact on Uruguay, including a rainfall increase [68]. *Galba*/*Fossaria* species, including both *L*. *viator* and *L*. *neotropica*, are amphibious snails which markedly depend on environmental and climatic factors such as temperature, water availability and evapotranspiration [2,7,9]. Thus, the predicted increase of rainfall [68] may *a priori* offer more possibilities for lymnaeid population growth and consequently the higher number and spread of vectors allow for an increased fascioliasis transmission.

The main aim of the present study is to assess the fascioliasis situation in Uruguay by means of the following aspects:

1. the genetic characterization of *F*. *hepatica* and the main lymnaeid vector species by nuclear ribosomal DNA (rDNA) and mitochondrial DNA (mtDNA) marker sequencing;
2. the assessment of the life cycle and transmission features of the Uruguayan fluke and lymnaeid in an infection experiment standardized regarding parasite, vector and abiotic factors, in the way to allow for significant comparisons;
3. a review of the human fascioliasis cases so far reported in the country.

The aforementioned results furnish the baseline needed for the understanding of the reasons underlying the difference between the high human prevalences and intensities in Andean highlands and the only sporadic human cases or small outbreaks in non-Andean lowlands. This purpose is achieved by means of the appropriate analysis of the following objectives:

1. the probable past origin/multi-origin of the fasciolids and main lymnaeid vector today present in Uruguay, by means of a correlation of the molecular results with historical data on the introduction and spread of livestock species into Uruguay;
2. a significant comparison of the disease transmission characteristics between the Uruguayan lowlands and the Bolivian Altiplano highlands presenting the highest human infection situation known;
3. the present and future risk of human infection in Uruguay by considering the genetic diversity of both parasite and main vector in front of the expected climate change predictions.

The latter objective becomes crucial for a country as Uruguay where cattle raising is the most important activity of the primary sector; cattle are kept on more than 83% of farms; on more than half of them beef cattle are the main source of income. The most important beef breed is Hereford, with 76.0% of the herd. There are more than 6,500 farms specialising in dairying, with more than 750,000 animals, more than 90% of which are Holsteins [69]. Moreover, Hereford and Holstein breeds appear to be the most affected by fascioliasis in Uruguay, with prevalences of 56% (95% confidence interval: 51–61%) and 68% (95% ID: 64–73%), respectively, pronouncedly higher than the prevalences in other breeds [41].

## Materials and methods

### Fasciolid and lymnaeid snail materials and Uruguayan livestock characteristics

Fasciolid materials were obtained from naturally infected animal hosts from Uruguay. A total of 46 fasciolid adult worms were obtained from livers of two Hereford, two Holando and two Aberdeen Angus breed cattle from three zones in the Salto department. Additionally, fasciolid eggs were obtained from a biliary filtrate of three horses from a slaughterhouse in Montevideo department (Fig 1). According to available facilities in obtaining materials, the collecting strategy was to sample materials from the western part of the country through which border the first fasciolids should have been introduced into Uruguay for the first time in the past, taking into account the original spread of livestock with the early Spanish conquerors. In Uruguay, fasciolids infect both cattle and sheep in the same places, because these two species are kept mixed in the grazing pastures and areas. Taking into account that *F*. *hepatica* infects cattle and sheep similarly (i.e., these two species do not select different fasciolid strains), the fasciolid material was obtained from cattle because this is the livestock regularly killed in the slaughterhouses, given that cattle raising is the most important activity of the primary sector in Uruguay. The material from horses was, however, not obtained in the same western areas, because horses also share the same pastures and should logically be infected by the same fasciolid haplotypes. Therefore, horses were selected from the Montevideo department, where these animals are managed in a somehow different way because of the neighbourhood of the big city.

Fasciolids from the aforementioned cattle and horses were used for DNA marker sequencing and a *F*. *hepatica* isolate obtained in a 6-year-old Hereford cattle female from Salto was used for the experimental infections of lymnaeid snails. Uruguayan fasciolid materials have been deposited in the Museu Valencià d’Història Natural, Alginet, Spain, under the code MVHN-241016MD01.

Lymnaeid snail materials originated from three different populations in Uruguay, corresponding to the departments of Montevideo (6 specimens), Paysandú (4 specimens) and Canelones (10 specimens) (Fig 1) and were used for their molecular characterization by DNA marker sequencing. Given the very low fasciolid larval stage prevalences in lymnaeid vectors in nature, a broader snail survey was not within the goals of this assessment of *F*. *hepatica* in Uruguay.

Moreover, lymnaeids collected in Canelones and shortly maintained in the laboratory of the DILAVE "Miguel C. Rubino", were finally transported and cultured in the Laboratory of the Valencia centre for a standardized experimental study. A total of 50 laboratory-borne specimens were used for the infection experiments with the aforementioned Uruguayan *F*. *hepatica* isolate. All lymnaeid specimens collected and used were preliminarily classified by shell morphology as *Lymnaea viator*, following the literature on lymnaeids in Uruguay (Table 1). Uruguayan lymnaeid materials have been deposited in the Museu Valencià d’Història Natural, Alginet, Spain, under the code MVHN-241016MD02.

**Table 1:** Uruguayan fasciolid and lymnaeid materials used, hosts, localities and DNA haplotype information for each molecular marker.

**Fasciolid materials**							
**Host species and breeds (individuals studied)**	**Locality**	**Fasciolid specimens haplotyped**	**DNA marker haplotype (H) codes**				
			rDNA ITS-2*	rDNA ITS-1*	mtDNA *cox*1*	mtDNA *nad*1*	**Combined H nomenclature**
Hereford cattle (n = 2)	Salto department	6 adult flukes	Fh-H1	Fh-HA	Fh-cox1-5	Fh-nad1-2	Fh1A,cox1-5,nad1-2
			365 bp, 48.49% GC	432 bp, 51.85% GC	1533 bp, 62.6% AT	903 bp, 65.3% AT	
			(KY048165)	(AJ243016)	(sequence in Fig 2)	(sequence in Fig 3)	
		4 adult flukes	Fh-H2	Fh-HA	Fh-cox1-5	Fh-nad1-2	Fh2A,cox1-5,nad1-2
			365 bp, 48.22% GC	432 bp, 51.85% GC	1533 bp, 62.6% AT	903 bp, 65.3% AT	
			(KY048166)	(AJ243016)	(sequence in Fig 2)	(sequence in Fig 3)	
Holando cattle (n = 2)	Salto department	3 adult flukes	Fh-H1	Fh-HA	Fh-cox1-42	Fh-nad1-12	Fh1A,cox1-42,nad1-12
			365 bp, 48.49% GC	432 bp, 51.85% GC	1533 bp, 62.6% AT	903 bp, 65.5% AT	
			(KY048165)	(AJ243016)	(sequence in Fig 2)	(sequence in Fig 3)	
		7 adult flukes	Fh-H2	Fh-HA	Fh-cox1-42	Fh-nad1-12	Fh2A,cox1-42,nad1-12
			365 bp, 48.22% GC	432 bp, 51.85% GC	1533 bp, 62.6% AT	903 bp, 65.5% AT	
			(KY048166)	(AJ243016)	(sequence in Fig 2)	(sequence in Fig 3)	
Aberdeen Angus cattle (n = 2)	Salto department	5 adult flukes	Fh-H1	Fh-HA	Fh-cox1-16	Fh-nad1-14	Fh1A,cox1-16,nad1-14
			365 bp, 48.49% GC	432 bp, 51.85% GC	1533 bp, 62.5% AT	903 bp, 65.4% AT	
			(KY048165)	(AJ243016)	(sequence in Fig 2)	(sequence in Fig 3)	
		5 adult flukes	Fh-H2	Fh-HA	Fh-cox1-16	Fh-nad1-14	Fh2A,cox1-16,nad1-14
			365 bp, 48.22% GC	432 bp, 51.85% GC	1533 bp, 62.5% AT	903 bp, 65.4% AT	
			(KY048166)	(AJ243016)	(sequence in Fig 2)	(sequence in Fig 3)	
Horse (n = 3)	Montevideo department	6 individualized eggs	Fh-H1	Fh-HA	Fh-cox1-42	Fh-nad1-12	Fh1A,cox1-42,nad1-12
			365 bp, 48.49% GC	432 bp, 51.85% GC	1533 bp, 62.6% AT	903 bp, 65.5% AT	
			(KY048165)	(AJ243016)	(sequence in Fig 2)	(sequence in Fig 3)	
		3 individualized eggs	Fh-H2	Fh-HA	Fh-cox1-42	Fh-nad1-12	Fh2A,cox1-42,nad1-12
			365 bp, 48.22% GC	432 bp, 51.85% GC	1533 bp, 62.6% AT	903 bp, 65.5% AT	
			(KY048166)	(AJ243016)	(sequence in Fig 2)	(sequence in Fig 3)	
Hereford cattle (n = 1)	laboratory strain for life cycle study	10 metacercariae	Fh-H1	Fh-HA	Fh-cox1-5	Fh-nad1-2	Fh1A,cox1-5,nad1-2
			365 bp, 48.49% GC	432 bp, 51.85% GC	1533 bp, 62.6% AT	903 bp, 65.3% AT	
			(KY048165)	(AJ243016)	(sequence in Fig 2)	(sequence in Fig 3)	
**Lymnaeid materials**							
**Preliminary classification**	**Locality**	**Classification after DNA characterization**	**DNA marker haplotype (H) codes**				
			rDNA ITS-2*	rDNA ITS-1*	mtDNA 16SrRNA**	mtDNA *cox*1**	**Combined H nomenclature**
*Lymnaea viator* (n = 6)	Montevideo department	*Lymnaea neotropica*	L.neo-H1	L.neo-HA	L.neo-16SA	L.neo-cox1e	L.neo-1A,16SA,cox1e
			417 bp, 56.83% GC	533 bp, 56.66% GC	429 bp, 69.47% AT	672 bp, 69.79% AT	
			(AM412225)	(AM412228)	(HE160433)	(KT215350)	
*Lymnaea viator* (n = 4)	Paysandú department	*Lymnaea neotropica*	L.neo-H1	L.neo-HA	L.neo-16SA	L.neo-cox1e	L.neo-1A,16SA,cox1e
			417 bp, 56.83% GC	533 bp, 56.66% GC	429 bp, 69.47% AT	672 bp, 69.79% AT	
			(AM412225)	(AM412228)	(HE160433)	(KT215350)	
*Lymnaea viator* (n = 10)	Canelones department	*Lymnaea neotropica*	L.neo-H1	L.neo-HA	L.neo-16SA	L.neo-cox1a	L.neo-1A,16SA,cox1a
			417 bp, 56.83% GC	533 bp, 56.66% GC	429 bp, 69.47% AT	672 bp, 69.94% AT	
			(AM412225)	(AM412228)	(HE160433)	(AM494008)	
*Lymnaea viator* (n = 50)	laboratory strain for life cycle study	*Lymnaea neotropica*	L.neo-H1	L.neo-HA	L.neo-16SA	L.neo-cox1a	L.neo-1A,16SA,cox1a
			417 bp, 56.83% GC	533 bp, 56.66% GC	429 bp, 69.47% AT	672 bp, 69.94% AT	
			(AM412225)	(AM412228)	(HE160433)	(AM494008)	

GenBank accession numbers in parentheses.

*, complete sequences;

++, partial sequences; bp, base pair

The living standard of Uruguay is closely related to earnings from pastoral and agricultural exports of beef and wool. Extensive cattle and sheep rearing is the main activity of Uruguay, where half the grassland is in estancias (usually large, simple buildings with thick walls, of a typical Spanish colonial style, with a lot of wrought iron) exceeding 2,000 acres. More than 13,500,000 ha are under permanent pasture, almost 83% of the agricultural area [69]. Millions of sheep and cattle are raised in the country. The preference for pasture over cropland is due to the excellence of the grasslands and the variable rainfall that makes grain production unreliable. The ratio between sheep and cattle production shifts with demand [70]. The predominant sheep breeds in Uruguay are Corriedale, Merino and Polwarth, which represent 60%, 20% and 10% of the national sheep flock, respectively. These breeds generate income from the sale of wool and sheep meat (surplus offspring and cast for age animals). Traditionally, wool has been the main product of the system. However, in recent years, the importance of sheep meat (lambs and mutton) has increased significantly [71].

### Molecular techniques

#### DNA markers

For the characterization of the fasciolid flukes, the sequence of the complete intergenic nuclear rDNA region, including the spacers ITS-2 and ITS-1 and the 5.8S gene, and the genes *cox*1 and *nad*1 of the mtDNA were selected. The usefulness of these markers for the molecular characterization of *Fasciola* species and strains has already been verified in a worldwide study [6].

For the characterization of the lymnaeid snails, the complete sequences of ITS-2 and ITS-1 and fragments of the mtDNA gene markers 16S rDNA and *cox*1 were used. These markers have already shown their usefulness for the classification of the lymnaeid vector species and the comparative assessment of the intraspecific variability of their populations in many countries of Latin America [32,33, 50,51,53,72–74].

#### DNA sequencing

Samples from fasciolid specimens (whether adult flukes and individualized eggs from natural infections or experimentally obtained metacercariae) from bovines and horses and snail head-foot tissue fixed in 70% ethanol were individually used for DNA extraction procedures (Table 1). Materials were suspended in 400 μl of lysis buffer (10 mM Tris-HCl, pH 8.0, 100 mM EDTA, 100 mM NaCl, 1% sodium dodecyl sulfate SDS) containing 500 μg/ml Proteinase K (Promega, Madison, WI, USA) and digested for 2 hr at 55°C with alternate shaking each 15 min. The procedure steps were performed according to methods outlined previously [48,50,75]. Total DNA was isolated according to the phenol-chloroform extraction and ethanol precipitation method. Each pellet was dried and resuspended in 30 μl sterile TE buffer (pH 8.0). This suspension was stored at –20°C until use.

Each one of the DNA markers was PCR amplified independently for each specimen and each PCR product was sequenced for a bona-fide haplotype characterization. The complete intergenic nuclear rDNA region including ITS-1, 5.8S and ITS-2 was amplified using primers previously described [48]. The complete *cox*1 and *nad*1 genes of fasciolids were obtained using forward and reverse primers designed in flanking regions of these genes [6]. The target fragments of the 16S and *cox*1 genes of lymnaeid snails were amplified by PCR using a set of universal primers [76,77]. Amplification procedures and thermal cycler conditions for each one of the DNA markers were carried out in a Mastercycle ep*gradient* (Eppendorf, Hamburg, Germany), as previously described [50].

PCR products were purified using the Ultra Clean PCR Clean-up DNA Purification System (MoBio, Solana Beach, CA, USA) according to the manufacturer's protocol and resuspended in 50 μl of 10 mM TE buffer (pH 7.6). The final DNA concentration was determined by measuring the absorbance at 260 and 280 nm on a Eppendorf BioPhotometer (Hamburg, Germany).

The sequencing of each molecular marker was performed on both strands by the dideoxy chain-termination method. It was carried out with the Taq dye-terminator chemistry kit on an Applied Biosystems 3730 DNA Analyzer (Applied Biosystems, Foster City, CA, USA) using PCR primers.

#### Sequence analyses

Sequences were aligned using CLUSTALW2 [78] in MEGA 6.0.6 [79] using default settings. Minor corrections for a better fit of nucleotide or indel correspondences were made in the cases of the ITS spacers. Homologies were performed using the BLASTN programme from the National Center for Biotechnology information website (http://www.ncbi.nlm.nih.gov/BLAST).

In the case of the snails, as the systematics of South American lymnaeids belonging to the *Galba*/*Fossaria* group is controversial and cannot be made with the single use of morphological criteria [33,50,51,53,74], for the specific classification of the lymnaeids, sequence comparisons were made using all available ribosomal and mitochondrial mollusc sequence data downloaded from GenBank.

#### DNA haplotype nomenclature

The haplotype (H) terminology used for the sequences obtained follows the standard nomenclature previously proposed for fasciolids [6] and lymnaeid snails [6,72]. It shall be noted that haplotype codes are only definitive in the case of complete sequences. When dealing with fragments or incomplete sequences, haplotype codes are provisional.

### Snail laboratory cultures

Living Galba/Fossaria lymnaeids preliminarily classified as *L*. *viator*, collected in Canelones and shortly maintained in the DILAVE laboratory, were transported under isothermal conditions to the laboratory of Valencia. Transfer to Valencia was needed to allow for a standardized laboratory adaptation and subsequent experimental follow-up of the life cycle and transmission of Uruguayan flukes by Uruguayan lymnaeids under abiotic conditions enabling significant comparisons with endemic areas of other countries. The possible natural infection by fasciolids was always individually verified prior to the launch of laboratory cultures. This was performed by keeping each lymnaeid specimen isolated in a Petri dish containing a small amount of natural water. After 24 h, the presence or absence of motionless metacercarial cysts or moving cercariae was verified in each Petri dish.

Afterwards, non-infected lymnaeids were arranged in standard breeding containers containing 2000 ml fresh water, to assure pure specific cultures. Finally, snails were adapted to and maintained under experimentally controlled conditions of 20°C, 90% relative humidity and a 12 h/12 h light/darkness photoperiod in precision climatic chambers (Heraeus-Vötsch VB-0714 and HPS-500). The water was changed weekly and lettuce added *ad libitum*.

### Liver fluke experimental infection assays

Eggs of *F*. *hepatica* from a 6-year-old bovine female from Salto were maintained in fresh water under complete darkness at 4°C until starting the embryogenic process. Embryogenesis was followed at 20°C at intervals of four days by counting eggs presenting an incipient morula, eggs including an advanced morula, eggs with outlined miracidium, and fully embryonated eggs containing a developed miracidium. Developed miracidia were forced to hatch by putting fully embryonated eggs under light and used for the experimental infection of snails [48].

Snails collected in the Uruguayan department of Canelones, shortly maintained in the laboratory of the DILAVE "Miguel C. Rubino", and finally transported and kept in the Laboratory of the Valencia centre were used for the experiments. Only laboratory-borne specimens were used. Snails of different size within the length range of 4.7–7.6 mm (mean 5.74 mm) were used to assess infection susceptibility. A total of 50 lymnaeid specimens were infected monomiracidially by exposing each snail to 1 miracidium for 4 hours in a small Petri dish containing 2 ml of fresh water. The disappearance of the miracidium was taken as verification of its successful penetration into the snail.

Snails were afterwards returned to the same standard conditions in the climatic chamber (2000 ml containers, 20°C, 90% relative humidity (r.h.), 12 h/12 h light/darkness, dry lettuce *ad libitum*) until day 30 post-infection, in which they were again isolated in Petri dishes to allow daily monitoring of cercarial shedding by individual snails. Lettuce was provided *ad libitum* to each snail in a Petri dish during both shedding and post-shedding periods until death of the snail. The chronobiology of the cercarial shedding was followed by daily counting of metacercariae in each Petri dish [48]. Furthermore, the strains of both *F*. *hepatica* and the lymnaeid used for the experimental assay were characterized by the sequencing of the aforementioned DNA markers. For that purpose, fasciolid metacercariae experimentally obtained and snails fixed after verification of the end of the cercarial shedding period were used (Table 1).

Life cycle aspects analyzed and respective methods used are in agreement with the standards applied for such studies in *Fasciola*. Following this standardized way allows for significant comparisons of the transmission characteristics in different endemic areas [48].

### Ethics statement

Ethical approval for the animal work was provided by the Ethics Committee for Animal Experimentation and Welfare of the University of Valencia, Spain (A1263915389140). Additionally, the División de Laboratorios Veterinarios (DILAVE), Montevideo, belongs to the corresponding national ministry (Ministerio de Ganadería, Agricultura y Pesca—MGAP) counting on its own ethics committee (Comité de Etica para Uso de Animales de Experimentación—CEUA) and its animal work is authorized by the Comisión Nacional de Experimentación Animal of Uruguay. Animal ethic guidelines regarding animal care strictly followed the institution’s guidelines based on Directive 2010/63/EU. Informed written consent was received from all animal owners (farm: El Solar, Salto; owners: Sucesores de Alfredo Sanchis; official registry No. at the MGAP Ministry: 150 606 365).

## Results

### DNA marker sequences of fasciolids

A total of 9 different marker sequences were obtained from the fasciolids. Nucleotide length of the sequences, their GC/AT content and reference codes are noted in Table 1.

Two haplotypes of the complete intergenic rDNA ITS1-5.8S-ITS2 region were detected in the fasciolids infecting cattle and also in horses in Uruguay. ITS-1 proved to have the same sequence in all specimens studied, corresponding to the haplotype A of this spacer (Table 1). ITS-2 showed two sequences in the Uruguayan fasciolids: haplotypes 1 and 2 GC (Table 1). Only one mutation in position 287 of the ITS-2 alignment allows the differentiation between both haplotypes: “C” in haplotype FhITS-2 H1 and “T” in FhITS-2 H2.

**Fig 2:**
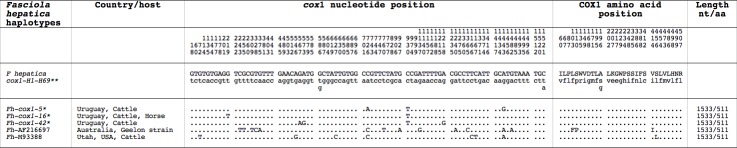
Differences found in mtDNA *cox*1 gene of *Fasciola hepatica* from Uruguay and other haplotypes. In the position where two or three different nucleotides or amino acids are noted, the first (noted with capital letter) is the majority one for *F*. *hepatica* intraspecific variation. Position = numbers (to be read in vertical) refer to variable positions obtained in the alignment made with MEGA 6.0.6;. = identical; * = present paper. ** 69 *F*. *hepatica* haplotypes described in Mas-Coma et al., 2009 [6] (39 populations from 10 countries); haplotype Accesion Number (Acc. No.) M93388 from Salt Lake City, Utah, USA of Garey and Wolstenholme, 1989 [82], and the haplotype Acc. No. AF216697 corresponding to the Geelon strain from Australia of Le et al., 2001 [83]. Nt = nucleotide base pair; aa = amino acids.

The mtDNA *cox*1 provided three different sequences with the same length. Their alignment showed 6 variable positions (all of them singleton sites). These sequences proved to enter in the intraspecific variability of the 69 *cox*1 haplotypes so far known in *F*. *hepatica*, corresponding to the haplotypes Fh*cox*1-5, Fh*cox*1-16 and Fh*cox*1-42 (Fig 2). The three haplotypes were found in cattle, whereas only Fh*cox*1-42 was found in horses (Table 1). The COX1 protein was 510 aa long, with start/stop codons of ATG/TAG, identical in all specimens analyzed, and corresponding to the haplotype FhCOX1-1 (Fig 2).

**Fig 3:**
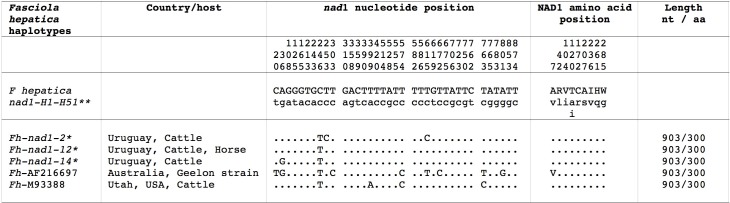
Differences found in mtDNA *nad*1 gene of *Fasciola hepatica* from Uruguay and other haplotypes. In the position where two or three different nucleotides or amino acids are noted, the first (noted with capital letter) is the majority one for *F*. *hepatica* intraspecific variation. Position = numbers (to be read in vertical) refer to variable positions obtained in the alignment made with MEGA 6.0.6;. = identical; * = present paper. ** 51 *F*. *hepatica* haplotypes described in Mas-Coma et al., 2009 [6] (39 populations from 10 countries); haplotype Acc. No. M93388 from Salt Lake City, Utah, USA of Garey and Wolstenholme, 1989 [82], and the haplotype Ac. No. AF216697 corresponding to the Geelon strain from Australia of Le et al., 2001 [83]. Nt = nucleotide base pair; aa = amino acids.

The mtDNA *nad*1 sequence provided three different haplotypes with the same length. Their alignment showed 3 variable positions (all of them singleton sites). These sequences also proved to enter in the intraspecific variability of the 51 *nad*1 haplotypes so far known in *F*. *hepatica*, corresponding to the haplotypes Fh*nad*1-2, Fh*nad*1-12 and Fh*nad*1-14 (Fig 3). The three haplotypes were found in cattle, whereas only Fh*nad*1-12 was found in horses (Table 1). The NAD1 protein showed only one 300-aa-long haplotype with start/stop codons of GTG/TAG in all specimens analyzed, corresponding to the haplotype FhNAD1-1 (Fig 3).

### DNA marker sequences of the lymnaeid snails

A total of 5 different marker sequences were obtained from the lymnaeids. Nucleotide length of the sequences, their GC/AT content and reference codes are noted in Table 1.

The ITS-2 sequences of lymnaeids from the three localities in Uruguay were identical. This unique sequence showed no one nucleotide difference with the ITS-2 haplotype H1 of the *Galba*/*Fossaria* species *L*. *neotropica* (Table 1). Similarly, the ITS-1 sequences were also identical in the three localities and without any difference when compared to the ITS-1 haplotype HA of *L*. *neotropica* (Table 1), which differs by two insertions in the “poli-A” region at the 3' end from haplotype HB (positions 512 to 529 including 16 or 18 consecutive “A” in L.neo-HA and L.neo-HB, respectively).

The 16S rRNA gene of the mtDNA provided only one haplotype in the three localities. This partial sequence presented a biased AT content, and proved to be identical to the provisional haplotype L.neo-16S HA (Table 1).

**Fig 4:**
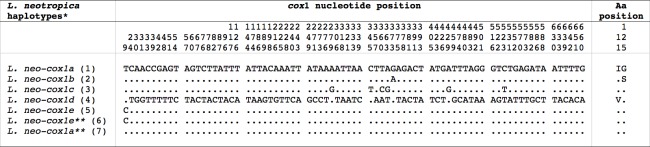
Differences found in mtDNA *cox*1 of *Lymnaea neotropica* from Uruguay and other haplotypes. Position = numbers (to be read in vertical) refer to variable positions obtained in the alignment made with MEGA 6.0.6;. = identical; ** = present paper. Haplotype codes only provisional due to incomplete sequences of the gene. *GenBank and country/locality noted in parentheses: 1 = AM494008 from Peru: Lima; Cajamarca; 2 = FN356741 from Argentina: Mendoza; 3 = JF461485 from Venezuela: Carabobo; 4 = JF461486 from Venezuela: Falcon; 5 = KT215350 from Argentina: Catamarca; 6 = KT215350 from Uruguay: Paysandu, Montevideo; 7 = AM49008 from Uruguay: Canelones.

The partial sequence of the mtDNA *cox*1 gene of lymnaeids from the three localities in Uruguay showed two haplotypes. The one found in Canelones proved to be identical to the haplotype L.neo-*cox*1 Ha from the type locality of *L*. *neotropica*. The second haplotype, present in the localities of Montevideo and Paysandu, proved to be identical to the provisional haplotype L.neo-*cox*1 He (Table 1). When comparing these two *cox*1 sequences from Uruguay with the five *cox*1 haplotypes of *L*. *neotropica* known so far, the resulting 672 bp-long alignment showed 76 variable positions, including two parsimony informative and 74 singleton sites. Nucleotide and amino acid differences are listed in Fig 4.

### Experimental life cycle

**Fig 5:**
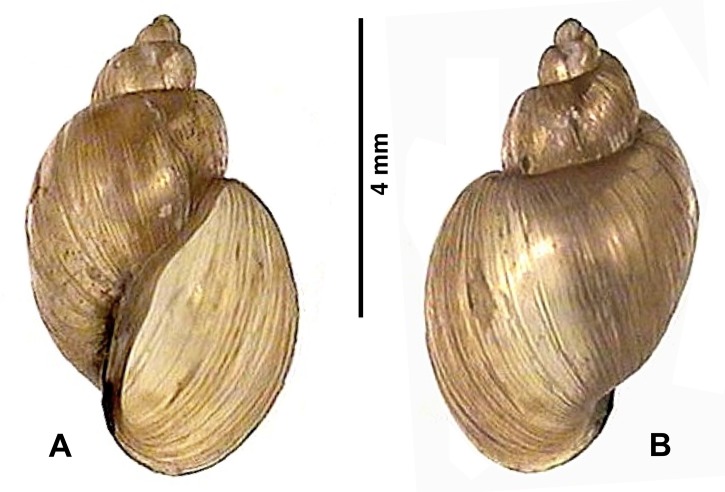
Shell of *Lymnaea neotropica* used for the experimental study of the transmission of Uruguayan *Fasciola hepatica*. **A.** Ventral view; **B.** Dorsal view.

Experimental life cycle studies were undertaken with the Uruguayan *F*. *hepatica* combined haplotype FhITS2-1, FhITS1-A, Fh*cox*1-5, Fh*nad*1-2 found in Hereford cattle from Salto, and the *L*. *neotropica* combined haplotype L.neo-ITS2-1, L.neo-ITS1-A, L.neo-16S-A, L.neo-*cox*1-a collected in the Canelones department (Fig 5).

Results of embryogenesis inside the egg, lymnaeid snail infection, intramolluscan parasite larval development and influences of the latter on snail survival are noted in Table 2. The use of identical experimental procedures and standardized abiotic factors allow for a significant comparison with the same data experimentally obtained with *F*. *hepatica* and *G*. *truncatula* from the high altitude pattern of the life cycle and disease transmission of cattle and sheep isolates of the liver fluke in the Northern Bolivian Altiplano, the human hyperendemic area with the highest human prevalences and intensities known (Table 2).

**Table 2:** Comparison of the results obtained in monomiracidial infection experiments of Uruguayan *Lymnaea neotropica* and Bolivian Altiplanic *Galba truncatula* with *Fasciola hepatica* isolates from Uruguay and the Northern Bolivian Altiplano, respectively.

Endemic area	Uruguay	Northern Bolivian Altiplano	
Epidemiological situation	Human infection in animal hyperendemic area	Human hyperendemic area	
Transmission pattern	Lowland pattern	High altitude pattern	
Experimental study	Present study	Mas-Coma et al., 2001 [48]	
Definitive host isolate	Cattle	Cattle	Sheep
Snail vector species	*Lymnaea neotropica*	*Galba truncatula*	*Galba truncatula*
Number of experimentally infected snails	50	25	32
Day presenting the maximum % of eggs with advanced morula	5 / 57.6%	8 / 86.7%	12 / 88.9%
Day in which the first outlined miracidium appeared inside egg	8	18	18
Day presenting the maximum % of eggs with outlined miracidium	14 / 37.3%	38 / 56.2%	36 / 37.3%
Day in which the first developed miracidium appeared inside egg	15	24	38
Day presenting the maximum % of eggs with developed miracidium	18 / 88.2%	46 / 24.9%	58 / 16.4%
Isolate infectivity (% snails infected)	74.5%	39.1	69.2
Prepatent period (days)	42–59 (49.2)	49–58 (51.0)	48–58 (49.6)
Shedding period (days)	1–19 (9.6)	42–85 (74.1)	47–88 (71.5)
No. cercariae shed per individual snail	4–1186 (269.2)	151–589 (446.2)	384–562 (451.8)
Snails surviving until shedding (% snails)	94.0	92.0	86.6
Snail survival after end of shedding period (days)	1–5 (1.3)	1–133 (55.4)	1–132 (44.5)
Postinfection longevity in shedding snails (days)	46–76 (59.2)	91–268 (165.0)	95–192 (157.1)
Longevity in non-infected snails (days post-infection)	16–56 (35.7)	100–209 (145.0)	98–182 (175.0)

Experiments performed under identical procedures and standardized abiotic conditions in precision climate chambers. Means in parentheses.

The Uruguayan liver fluke isolate proved to follow a pronouncedly faster embryogenesis. The first developed miracidium appears already in day 15, with the maximum percentage of eggs including fully developed miracidia in day 18, whereas 46 and 58 days were needed by cattle and sheep isolates from the Bolivian Altiplano, respectively. Such a development speed is three times faster in the lowlands of Uruguay, even in a surprisingly high percentage of eggs of 88.2% (whereas only in 24.9% and 16.4% for the two Bolivian isolates, respectively) (Table 2).

The high snail infectivity rate (74.5%) of the Uruguayan isolate is worth mentioning, although similar to the Bolivian sheep isolate. The prepatent period (days elapsed from infection day up to the first day of cercarial shedding) in the Uruguayan isolate is markedly similar to that of the two Bolivian isolates. However, pronounced differences appear in the shedding period (total number of days in which the snail was shedding cercariae), as well as in the total number of cercariae (and subsequent metacercariae) produced by each infected lymnaeid. The shedding period in the Uruguayan isolate proved to be very short, of only 1–19 days (mean 9.6 days), despite of which the number of cercariae per snail was relatively high, of 4–1186 cercariae/snail (mean 269.2). These features are, however, far from the ones characterizing the Altiplanic liver fluke isolates, in which the shedding period is very long (averages higher than 70 days in both isolates) and the number of cercariae per snail is very high (averages higher than 445 cercariae/snail in both isolates) (Table 2).

The geographical isolate did not seem to influence lymnaeid survival during the prepatent period, results obtained with the Uruguayan fluke being very similar to those in the two Bolivian isolates. Nevertheless, (i) the snail survival after the end of the shedding period, (ii) the postinfection longevity in shedding snails, and (iii) the longevity in non-infected snails, were all three pronouncedly shorter when dealing with the Uruguayan isolate than with the two Bolivian isolates (Table 2).

**Fig 6:**
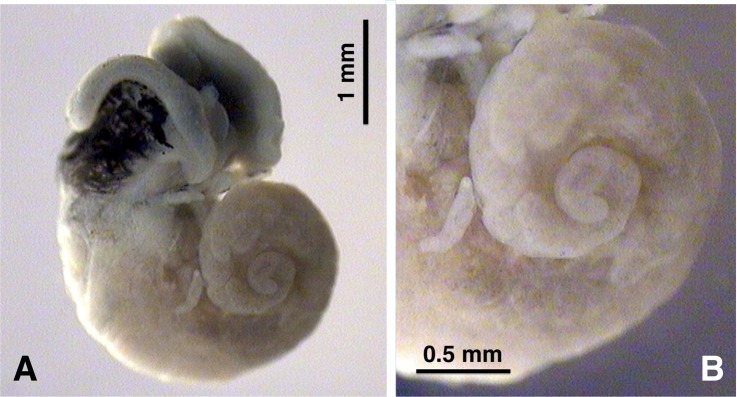
Soft parts of *Lymnaea neotropica* infected with *Fasciola hepatica*, at the end of cercarial shedding. **A.** Apical view showing extent of redial infection. **B.** Magnification showing massive presence of rediae.

Despite the fast and short intramolluscan larval development, the Uruguayan liver fluke isolate proved to be able to reach a marked extent of redial infection and massive presence of rediae in the local Uruguayan lymnaeids (Fig 6A and 6B).

### Chronobiology of the cercarial emergence

**Fig 7:**
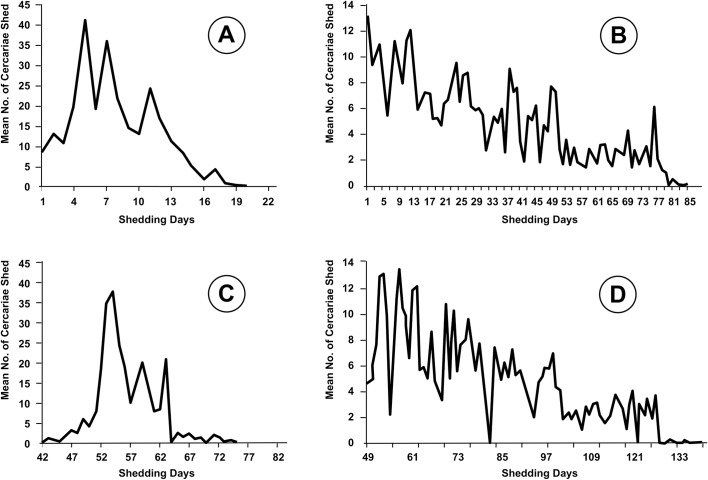
Cercarial shedding of *Fasciola hepatica* by *Lymnaea neotropica* from Uruguay and by *Galba truncatula* from Bolivia. Studies on the fluke/snail couple from Uruguay (A, C) and from the Northern Bolivian Altiplano (B, D) performed under identical procedures and standardized experimental abiotic factors [48]. **A, B.** Shedding period analyzed from the day of the emergence of the first cercaria by each snail. **C, D.** Shedding period analyzed from the day of the miracidial infection.

The chronobiological pattern of cercarial emergence in the cattle isolate of *F*. *hepatica* is shown in (Fig 7A and 7C).

When the shedding period is analyzed from the day of the emergence of the first cercaria by each snail (Fig 7A), the shedding process appears as an irregular succession of waves. After four days of an initial shedding of a reduced daily number of cercariae, a slow decrease of that number is observed. The higher acrophases take place at the end of the first week and during the second week.

When the shedding period is analyzed from the day of the miracidial infection (Fig 7C), most of the cercariae are shed between days 52 and 64 post-infection (p.i.). The days 64 and 73 p.i., in which all snails failed to shed any cercaria, suggest an intramolluscan larval development including up to a maximum of three redial generations. It is mainly for the first generation to produce and shed most of the cercariae.

When comparing this chronobiological pattern of cercarial emergence in *F*. *hepatica*/lymnaeid snail from Uruguay (Fig 7A and 7C) with the chronobiological pattern in *F*. *hepatica*/*G*. *truncatula* from the Northern Bolivian Altiplano, performed under identical procedures and standardized experimental abiotic factors (Fig 7B and 7D), four main differences should be highlighted:

1. in the Uruguayan fluke/lymnaeid couple, the daily number of cercariae per snail is high, with acrophases showing mean numbers between 20 and 40 (Fig 7A and 7C), markedly higher than the acrophases not even reaching mean numbers of 14 in the Bolivian fluke/lymnaeid couple (Fig 7B and 7D).
2. in the Uruguayan couple, the cercarial emergence appears concentrated in less than 20 days (throughout a period of only 30 days p.i.) (Fig 7A and 7C), whereas it follows a very long cercarial emergence process of up to 85 days, well synchronized in all shedding snails, in the Bolivian couple (Fig 7B and 7D).
3. in the Uruguayan couple, the daily number of emerging cercariae gradually increases during the initial 5–6 days until reaching the highest acrophase (Fig 7A), whereas this number appears to be directly the highest at the starting day of the shedding to afterwards decrease gradually, in the Bolivian couple (Fig 7B).
4. in the Uruguayan couple, second and third cercariogenous redial generations have a negligible contribution to the cercarial shedding, given the very small number of cercariae emerging after day 64 p.i. in which all snails failed to shed any cercaria (Fig 7C), whereas the second redial generation (between days 83 and 122 p.i.) pronouncedly contributes, both quantitatively and timely, to the cercarial shedding (less the third generation) in the Bolivian couple (Fig 7D).

## Discussion

### Molecular characterization of *Fasciola hepatica* of Uruguay

Fasciolids from Uruguay molecularly prove to belong to widespread strains of *F*. *hepatica*, fitting within the intraspecific variability of this fasciolid species in Europe and Latin America. Indeed, *F*. *hepatica* was introduced into South America throughout a process which started 500 years ago at the time of the first Spanish colonizers, who were transporting livestock in their ships [6]. From the evolutionary point of view, such a period is very short for a parasite. Manter's parasitophylogenetic rule, about the slower evolution of parasites when compared to that of the hosts, should be considered here [80]. Moreover, the livestock host species in Latin America at present are the same than in its original spreading area in Europe 500 years ago, which means that the microhabitat of *F*. *hepatica* has not changed despite its hosts having been moved from one continent to another. Additionally, the evolutionary rates of the four DNA markers used are too low [81] as to expect mutations appearing by isolation in the Americas and differentiating American fasciolids from those of the Old World [6].

Short information may be inferred from the only one ITS-1 and two ITS-2 haplotypes of these evolutionarily conserved rDNA spacers. In fact, the worldwide spread of *F*. *hepatica* occurred only during the last 12,000–10,000 years, from the moment of the domestication of livestock in the Fertile Crescent in the Near East and in Old Egypt, when humans began to expand livestock species throughout. Similarly occurred with *F*. *gigantica*, although restricted to Africa and Asia where its specific *Radix* lymnaeid species are present. The absence of *Radix* in the New World (only isolated populations of *R*. *auricularia* introduced into North America from Europe) explain why only *F*. *hepatica* is present in the Americas [6]. A period of 12,000–10,000 years is too short for ITSs to give rise to mutations, given their evolutionary rate [81]. Thus, in regions where only *F*. *hepatica* is present, and consequently there is no possibility for hybridization with *F*. *gigantica*, the same only FhITS-1 HA is known [6]. Regarding ITS-2, FhITS-2 H1 and FhITS-2 H2 found in Uruguay also correspond to the two haplotypes known in areas presenting genetically pure *F*. *hepatica* [6]. FhITS-2 H1 is the most widely distributed, whereas FhITS-2 H2 has interestingly been described from Spain and Andorra [6] and was already previously reported from Uruguay [82].

More information can be inferred from the three *cox*1 and three *nad*1 haplotypes of the faster evolving mtDNA [81]. Among *cox*1, Fh*cox*1-5 and Fh*cox*1-16 are widely dispersed in South America, but Fh*cox*1-42 has only been found in Bolivia [6]. A similar picture is provided by *nad*1. Fh*nad*1-2 and Fh*nad*1-14 are distributed throughout North and South America, whereas Fh*nad*1-12 has only been detected in Peru, Bolivia and Argentina [6]. The only two other complete mtDNA gene sequences of *F*. *hepatica* from Salt Lake City, Utah, USA [83] and the Geelon strain in Australia [84] are different from the *F*. *hepatica* haplotype group of the Iberian Peninsula and Latin America, at the level of both *cox*1 (Fig 2) and *nad*1 (Fig 3).

The single COX1 and NAD1 protein haplotypes found in Uruguay are the most abundant, both widely distributed in different countries and different host species [6].

The detection of Fh*cox*1-42 and Fh*nad*1-12 in both cattle and horses indicate infection from same sources, e.g. in Uruguay horses may become infected when grazing in pastures used for cattle and sheep [44].

### Classification and molecular characterization of *Galba*/*Fossaria* lymnaeids of Uruguay

The sequences of the four DNA markers used unambiguously demonstrate that the *Galba*/*Fossaria* lymnaeids collected in the three Uruguayan localities belong to the species *L*. *neotropica*.

The ITS-2 and ITS-1 found in Uruguay are identical to L.neo-ITS-2 H1 and L.neo-ITS-1 HA in Perú and Argentina (74,50,51,75), the latter differing by only two "A" insertions from L.neo-ITS-1 HB from Argentina [51]. Similarly, the 16S haplotype L.neo-16S HA found in Uruguay has also been reported from Perú and Argentina [50,51]. The first *cox*1 haplotype L.neo-*cox*1 Ha found in Canelones was already known from the type locality of *L*. *neotropica* and another area in Peru [50,74]. The second L.neo-*cox*1 He found in Montevideo and Paysandu was known only from Argentina [51].

So far, the only *Galba*/*Fossaria* species reported in Uruguay was *L*. *viator* [36,55,56,59–66]. Consequently, *L*. *neotropica* becomes a new species for the Uruguayan fauna. Its finding in areas located far away one another, indicate that this species should be widely distributed throughout the country. The question posed now is whether both *L*. *neotropica* and *L*. *viator* coexist in Uruguay, or there is simply only *L*. *neotropica* which has always been confused with *L*. *viator*.

Indeed, DNA sequencing of lymnaeids started at the end of the last century, including markers of mtDNA [85] and nuclear rDNA [86]. Their progressive use highlighted the problems in specimen classification and species differentiation by traditional malacological approaches [48,72,73]. Interspecific differentiation in *Galba*/*Fossaria* became the main focus, due to their importance in the transmission of *F*. *hepatica*. The description of the new species *L*. *neotropica* and its molecular differentiation from *L*. *viator* and *G*. *truncatula* was an important step forward [74]. The molecular demonstration that the hitherto overlooked species *L*. *schirazensis*, without fascioliasis transmission capacity, had always been confused with *G*. *truncatula* and other *Galba*/*Fossaria* vector species, illustrated up to which level there was a chaotic systematic situation [53], as verified in Venezuela [33]. Similarly, DNA sequencing proved the presence of *G*. *truncatula*, *L*. *neotropica* and *L*. *schirazensis* in the human fascioliasis hyperendemic area of Cajamarca, in Peru, where only *L*. *viator* had been involved [50].

All these major advances were posterior to all studies on lymnaeids in Uruguay. This suggests that we are only dealing with a classification confusion between *L*. *neotropica* on one side and *L*. *viator* and *G*. *truncatula* on the other side, similarly as happened in Peru, Venezuela, Argentina, and also Chile [30]. So, *L*. *neotropica* should probably be the only *Galba*/*Fossaria* species distributed throughout Uruguay. However, it should not be overlooked that *L*. *neotropica* and *L*. *viator* may coexist in the same area, as in Mendoza [87,88] and Catamarca [51], both in Argentina. Studies in other areas of Uruguay are needed. For this purpose, it shall be considered that ITS-1 and 16S showed the highest and lowest resolution for interspecific differentiation, respectively, whereas *cox*1 was the best marker and ITS-1 the worst for intraspecific analyses [51].

Regarding genus ascription of *L*. *neotropica* and *L*. *viator*, their last molecular comparison, both one another and inside the *Galba*/*Fossaria* group, and maximum support values obtained for the internal branching nodes in the phylogenetic analysis of the species of *Galba*/*Fossaria*, demonstrated that these Neotropical species do not belong to the genus *Galba* defined by its Palaearctic type species *G*. *truncatula* [51]. Until sequence data from the very large number of *Galba*/*Fossaria* species known from the Nearctic region [89,90] is obtained, caution recommends to taxonomically keep *L*. *neotropica* and *L*. *viator* in the genus *Lymnaea sensu lato*.

The sequences of *L*. *neotropica* from Uruguay and those from Peru [50,74], Venezuela [33] and Argentina [51,75,88], suggest that the spread of this lymnaeid throughout the Neotropical region should have occurred very recently, passively transported with livestock exchange, in a similar way as other *Galba*/*Fossaria* species spread throughout even different continents, as *G*. *truncatula* [6] and *L*. *schirazensis* [53].

Finally, it should be highlighted that all the haplotypes of the four DNA markers found in *L*. *neotropica* from Uruguay have also been found in two human fascioliasis endemic areas, such as Cajamarca in Peru [50] and Catamarca in Argentina [51].

### Correlation of fasciolid and lymnaeid haplotypes with historical data

#### Baseline

Present-day Uruguay was discovered by the Portuguese in 1512, the Spanish arriving in 1516. Physically, Uruguay is both a continuation of the Argentina Pampas and an extension of the Brazilian shield. The dominant vegetation in the colonial period was tall grasses, and more than 70% of Uruguay remains in pasture [70].

Both *F*. *hepatica* and the two lymnaeids *L*. *neotropica* and *P*. *columella* were introduced by humans into Uruguay. *Fasciola hepatica* is known to be of Palaearctic origin and was initially introduced into the Americas by the Spanish colonizers when transporting European livestock infected by the liver fluke [6]. The original geographical distribution of *Lymnaea neotropica* should be looked for in the northern Andean region where it shows the highest DNA variability ([33,50,74] and, as other amphibious *Galba*/*Fossaria* species, should have been introduced into Uruguay together with foreign livestock in dried mud stuck to the feet of the animals [6,53]. *Pseudosuccinea columella* is original from the region of Central America and the circum-Caribbean area [32,33] and may also have been introduced similarly, although most probably attached to transported vegetables given its ethological characteristics [33].

**Fig 8:**
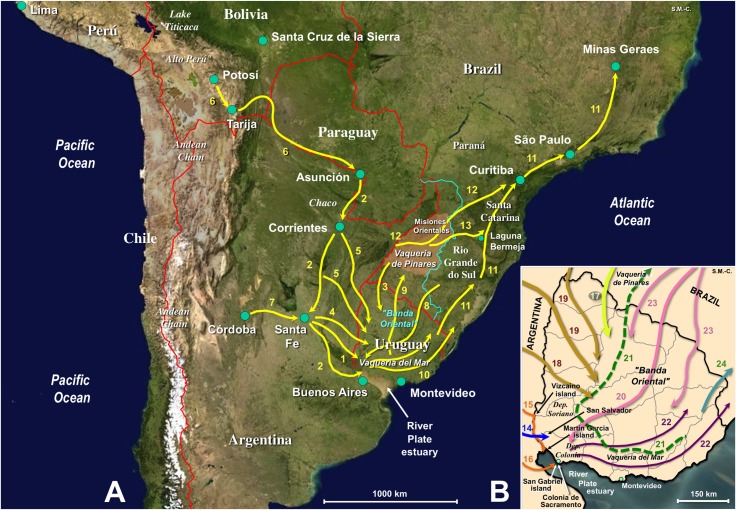
Livestock movement timeline in the early introduction and spread of *Fasciola hepatica* and lymnaeids in Uruguay. Maps of South America (**A**) and Uruguay (**B**) showing main livestock passageways during the early Spanish and Portuguese colonizations. The analysis concerns the time of the old Viceroyalty of Rio de la Plata, from Buenos Aires in the South and the "Banda Oriental" in the Southeast up to "Alto Peru" in the North. 1, 14, First introductions of pigs in 1541, horses in 1574, and goats in 1577; 2, 15, 16, First and second introduction of cattle derived from Corrientes population in 1611 and 1617; 3, 17, Introduction of cattle from Misiones by Jesuits at the beginning of 17th century; 4, 5, 18, 19, First introduction of sheep from Santa Fe in 1727 (4, 18) and subsequent large scale cattle introductions with "faeneros" from Asuncion, Corrientes and Santa Fe (4, 5, 18, 19); 6, Livestock route for silver transport from Potosi mines from mid 16th century; 7, Original route for introduced goats in 1611–1618; 8, 20, Introduction of sheep by the Portuguese in 1734–1735; 9, 21, Largest rustle of more than 400,000 cattle in 1705, from Vaqueria del Mar to Vaqueria de los Pinares, at the southern part of the Jesuit Misiones Orientales area (brownish area); 10, 22, Livestock spread at mid and end of 17th century; 11, Livestock route (Camino Real, Ruta del Viamont or Caminho do Viamão) for gold transport from Minas Geraes mines from 1690; 12, Interconnection livestock route (Ruta de las Misiones or Caminho das Missões); 13, Interconnection livestock route (Ruta de la Vaquería or Caminho da Vacaria); 23, Groups of Portuguese "bandeirantes" also using livestock; 24, Northward spread of livestock. Background for A from composed satellite map of South America orthographic projection by NASA (full resolution of 1,215 x 1,712 pixels; public domain) via Wikimedia Commons. Original S. Mas-Coma.

A map showing the timeline information on historical livestock movements noted in the following sections is included in Fig 8.

#### First introductions of livestock species

Equines were the first introduced into Uruguay. The first horses were introduced into the northern part of the department of Colonia, less probably in the south of the department of Soriano, in 1574 and originated from Asunción, Paraguay, although transported from Santa Fe, Argentina [91]. Native indians probably used them until a second introduction of several horses (together with cattle) from the northern coast of the River Plate region into the Uruguay riverbank, first in 1611 and again in 1617 [69]. Subsequently, horses quickly multiply [92]. In 1596 the commercial trade of mules started to work [93]. A few horses were noted in Colonia de Sacramento in 1680 [90], and around 18,000 equines, including horses, mules and donkeys, were already present in Colonia del Sacramento in 1735 [94]. The original Creole horses were created when mixing with different English races in the mid-XIX century.

American Creole cattle descend from Iberian breeds introduced by the Spanish and Portuguese conquerors in the 15th century [95]. The first bovines were introduced in Vizcaino island in 1611 and on the shores in front of San Gabriel island in 1617 [96]. Around 100 cattle and some horses from Argentina were shipped and landed on the Uruguay riverbank where they were left to run wild [69]. These animals derived from cattle existing in Corrientes, Argentina, where they were introduced from Asunción. These cattle breed originated from the Peruvian highlands after having been transported through the Bolivian valleys around Tarija, afterwards the lowlands of Santa Cruz de la Sierra and finally through the Paraguayan Chaco up to Asunción in 1568 [91]. Jesuits probably introduced cattle contemporaneously (or shortly later, when not even previously). They led the cattle they had in their missions in Paraguay and upper River Uruguay [97] to graze in the prairies between the River Uruguay and the Atlantic Ocean (northern shore of the River Plate) [91,96]. The first cattle spread at large scale in Uruguay took place in 1634, when 1500 animals were transported from Corrientes up to the at present Brazilian state of Río Grande do Sul. Among several animal transportation initiatives at the beginning of the following century, the largest rustle was in 1705 and included more than 400,000 cattle which were driven with many horses from the southern Vaquería del Mar up to the northern Vaquería de los Pinares, at the south of the at present Brazilian state of Santa Catarina [91].

The cattle introduced at the beginning of the 17th century multiplied immediately: thousands first, multitudes ("millions"?) later, becoming semi-wild and roaming over the territory known later as the Banda Oriental of the River Uruguay [98]. Thus, "gaucho" horsemen culture and pastoral life became the way of life for the European colonizers that would later populate the region. This way of life lasted throughout the following three centuries [96]. In the 1770s the process of dividing the Banda Oriental into huge unfenced "estancias" began [69]. By 1880 much of Uruguay was subdivided into cattle "estancias".

The introduction of "Durham" cattle from England In 1859 was the first step of a sustained importation trend of different pure cattle strains henceforth. This led to a progressive mixing of the Creole cattle, which were slowly substituted by the new races with higher adaptation and reproduction capacities [99]. By the 1890s, Hereford and Shorthorn cattle were upgrading the quality of Uruguayan beef. Thus, the huge initial population of Creole cattle was reduced to small and sparse subpopulations throughout the country [95].

The first introduced sheep, probably in 1727, were of the Spanish "Churra" strain coming from Peru through Asunción and Santa Fe. A few sheep in 1735, and more than 2000 in 1737, were reported in Colonia de Sacramento. Around 8200 sheep are noted to exist in Montevideo in 1751–1752. These reports suggest that Portuguese colonizers were those who launched the sheep breeding [91,92]. Other sheep breeds were subsequently introduced to improve the poor "churra" quality, such as the Spanish "Merino" from Cadiz in 1792, the "Merino of the French type of Naz" in 1838, the strains "Sajona Negrette" and "South Down" in 1840, the " Romney Marsh" and the "Merino Mauchamps" in 1854, the "Ramboulliet" in 1855, and the "Lincoln" in 1862 [100]. By the end of the 1860's decade the introduction of sheep modernized and capitalized Uruguay's livestock farming system, with wool overtaking leather and beef as the primary export commodity by the 1880's [101]. In the mid 19th century there were 662,500 sheep of the “Criolla” breed and 133,700 crosses. By 1860 sheep rearing was strongly promoted. After 1870, Uruguay had more sheep than cattle, largely because of an influx of sheep ranchers from France and Britain [69]. Additional sheep introductions followed in the XX century, including the "Corriedale" breed from New Zealand in 1912, the "Ideal" breed from Australia in 1913, the "Merino" breed of Asian origin, the "Hampshire Down", "Suffolk" and "South Down" strains from England in 1940, and the "Texel" strain from The Netherlands in 1972 [102].

Goats are first reported from San Salvador, Uruguay, in 1577. Specimens from Cordoba, Argentina, were introduced into the islands of San Gabriel and Martín García in the 1611–1618 period [91]. However, this species was never given special care [99], nor used for production in the country. At present goat numbers are only around 8,000, among which 3,000 are for milk and kid production [69]. Pigs were first introduced in 1541 in San Gabriel island and also mentioned in San Salvador in 1577 [91], but did never become important.

#### Livestock mixing through historical corridors and recent management

The big silver production of the mines in Potosi, in the High Peru ("Alto Peru", today Bolivia), from the mid 16th century, and later in Minas Geraes, Brazil, where gold was discovered in 1690, requested new routes for mineral and good transport. The Banda Oriental of Uruguay pronouncedly benefitted from that situation by providing cattle and mules and also horses. These routes took advantage of the large internal corridors of the Argentinian valleys used by the native indians in the past and the lowlands of Uruguay (the so-called "Oriental Corridor") connecting with the present southern Brazil [93]. During that period, Uruguay was providing livestock (cattle and sheep, but also mules) to Buenos Aires directly by ship [103] and also by land through Santa Fe, from where animals were also transported to Peruvian highlands [104]. On the Portuguese side, the foundation of Colonia de Sacramento in southwestern Uruguay, at the Rio de la Plata estuary shore just opposite to Buenos Aires, in 1680, lead to open a northward route to the Brazilian localities of Laguna and Curitiba in 1722 and subsequently to Sao Paulo and Minas Geraes in 1731 [105]. Around 1780, the administrative area of Montevideo and its harbour were integrated by land to the corridors of the Argentinian valleys and southern Brazil and by maritime way with Europe, thus completing a network covering the whole Banda Oriental of Uruguay [93].

Over wide tracts of Uruguay, a very common characteristic of livestock production is mixed grazing, particularly beef cattle with sheep for wool. The complementarity of cattle/sheep began with the sheep expansion in the 19th century. A farmer could make a better use of his farm and, in addition, the sensitivity of both species to climate was balanced. High rainfall affects cattle less and sheep tolerate droughts better [69].

At present, cattle movement networks are characterized by lower levels of connectivity and higher levels of heterogeneity than random networks of the same size and density. The majority of farms have few to no contacts, whereas the 10% most highly connected farms account for 72–83% of animals moved annually. This extreme level of heterogeneity in movement patterns indicates that some farms may be disproportionately important for pathogen spread [106].

Approximately 380,000–400,000 equines are used for monitoring and herding of animals, and shall therefore also be considered. Only a few are used for draught or recreation. Around 35,000 cull horses are annually slaughtered only for export [69].

#### Correspondence of DNA results with livestock history

Concluding statements on *F*. *hepatica* and lymnaeid vector introduction/arrival into, and spread throughout, Uruguay in the past can be inferred from: (i) the distribution of the Uruguayan fasciolid and lymnaeid haplotypes in neighbouring countries as evidence of former geographical exchange; (ii) the livestock movement timeline established on the background of the aforementioned information furnished by historical records. DNA marker sequences obtained from both *F*. *hepatica* and *L*. *neotropica* from Uruguay fit well with the aforementioned scenario about the past and present of livestock in this country. Although the parasite may arrive together with the animal reservoirs, the disease cannot be introduced and established unless there is already a vector species present in the newly colonized area.

It may be concluded that *L*. *neotropica* was fundamental in both introduction and spread of fascioliasis, given the grazing lowlands and its crucial role in the disease transmission in Uruguay. Indeed, this lymnaeid species had several occasions to be introduced, so that more than one introduction should not be ruled out. Already the first introduced cattle in 1611–1617 were from a breed originated from Corrientes and Asunción in its turn derived from animals of the Peruvian highlands. Moreover, in these early times cattle were brought from the Jesuit missions in Paraguay to graze in the Uruguayan prairies, and further cattle spread eastwards into Uruguay and even southern Brazil was already practiced from 1634. Sheep may also have participated in the *L*. *neotropica* introduction, as the first introduction of "Churra" sheep in 1727 were indeed coming from Peru through Asunción and Santa Fe. The quick spread of sheep throughout Uruguay in the following decades may also have had a prominent role in the expansion of this lymnaeid in the country. Later on, the spread of cattle grazing and the promotion of sheep rearing after 1860 probably contributed to this process. This fits well with the DNA marker haplotypes of Uruguayan *L*. *neotropica* which are identical to those previously found in Peru and Argentina. This suggests the passive introduction of this lymnaeid by cattle, sheep or both. The first reports of a *Galba*/*Fossaria* lymnaeid, ascribed to *L*. *viator*, date from the end of the 19th century [55] and beginning of the 20th century [56]. In 1916, this species was found in a marsh close to the river Uruguay [107]. Many years later, infected *L*. *viator* specimens were found in the river Santa Lucía, at only 80 km distance from Montevideo [59].

Present knowledge on DNA sequences of *P*. *columella* indicate that this lymnaeid undertook its spread throughout all continents from its original geographical distribution in Central America, southern North America, northern South America and the Caribbean only very recently [33]. Thus, the presence of *P*. *columella* in Uruguay should most probably be considered the result of a human introduction in more recent times, similarly as in whole South America. The first report of *P*. *columella* in Uruguay dates from the end of the 19th century [57]. Given its present widespread distribution throughout the southern states of Brazil [108], it may logically be deduced that it was introduced thanks to the transport of goods carried out and/or livestock (cattle, mules) used through the south-north corridor between Colonia de Sacramento and the Brazilian states during the period since 1722.

DNA haplotypes indicate the present existence in Uruguay of *F*. *hepatica* populations resulting from the mixing of populations from different geographical origins and livestock species. Indeed, liver fluke isolates from different mammal species show similar capacities of infecting other mammal species [109,110]. The Iberian Peninsula appears to be the most important origin, whether from Spain through previous transits in other South American neighouring countries or from Portugal through southern Brazil. The introduction of more selected European breeds of both cattle and sheep, mainly of English origin, but also from other continents (e.g. Oceania, Asia) from the mid-19th century may have also contributed to the introduction of other *F*. *hepatica* DNA haplotypes. The presence of the same Uruguayan haplotypes in countries very far away, such as Poland or Mexico, speak about such late introductions. Present day exchange of livestock between highly connected farms may have a further role in *F*. *hepatica* haplotype spread throughout Uruguay in recent years. The capacity for hybridization by crossing in this hermaphroditic trematode [6] adds complexity to its already wide geographical mixing in this country.

### Fascioliasis transmission capacity

Uruguayan *F*. *hepatica* and *L*. *neotropica* used for the experiments were selected to assess the disease transmission capacity of the most common and widely dispersed strains of both fluke and vector species.

The embryonation time found in the Uruguayan couple proves to be very short. It fits within the fastest inside the range known when tested at 20°C [111–113]. In *F*. *hepatica*, the development of the miracidium inside egg is arrested below 9°C and above 37°C and has a duration between 9 and 161 days depending upon the temperature, the range 20–25°C offering the optimum for the hatching of a higher number of miracidia [114–117].

The detected infection percentages and prepatent period in monomiracidial infections may be considered as normal at 20°C when compared to similar studies carried out with *F*. *hepatica* isolates and *G*. *truncatula* specimens from European areas [111,114,118–122]. The prepatent period found in the Uruguayan couple also fits in the known range (43.1 ± 58.2 days) for European *F*. *hepatica*/*G*. *truncatula* in the nature [123].

The shedding period in Uruguayan *F*. *hepatica*/*L*. *neotropica* is very short. In European *F*. *hepatica* experimentally infecting *G*. *truncatula* snails of the same size as ours under the same constant conditions of 20°C and 12 h/12 h photoperiod, the patent period lasted only 46 ± 27.6 days [124]. Similarly, results obtained in nature show that the patent period in Europe ranges between 5.0 and 9.3 days in the winter generation and 18.3–40.3 days in the summer generation [123]. The pronounced differences of the very short shedding period in the couple from the Uruguayan lowlands when compared to the very long one in the *F*. *hepatica*/*G*. *truncatula* couple from the highlands of the Bolivian Altiplano (Table 2) [48] should be highlighted.

The mean number of cercariae shed per individual lymnaeid in the Uruguayan couple is close to the mean of 238.5 cercariae/snail found in the European *F*. *hepatica*/*G*. *truncatula* model under the same experimental conditions [124]. A lower number of 114.9 ± 80.3 cercariae per monomiracidially infected snail were obtained with the same European couple. These experimental assays showed that the duration of the shedding and the number of cercariae were independent of the number of miracidia used for the infection of each individual lymnaeid. However, single-miracidium infections were most effective because of the much higher snail survival rate, despite the mean number of cercariae shed being the same as in multimiracidial infections [125]. However, the most important is the marked difference when compared to the pronouncedly higher mean cercarial numbers in the *F*. *hepatica*/*G*. *truncatula* couple from the Bolivian Altiplano (Table 2) [48].

Differences in survival of different geographical strains of the same *Galba*/*Fossaria* species to *F*. *hepatica* infection have already been described [126]. The postinfection longevity in shedding *L*. *neotropica* from Uruguay is only slightly shorter than the 70 days p.i. usually observed in European *G*. *truncatula* [127–131], with a maximum of 16 weeks described once [128,129], and far from that known in other American lymnaeids such as 119 days p.i. for *L*. *viator* [132] and 113.4 days p.i. for *L*. *bulimoides* [133]. A fast development and extensive massive infection of the larval stages in *L*. *neotropica* (Fig 6) may be the responsible for a quick snail mortality, similarly as in European *G*. *truncatula*. It was found that of 102 snails shedding on the first day, the number drastically reduced to only 56 on the second day and subsequently decreased on day 76 to four snails [124]. Regarding this aspect, the pronounced difference when compared with the capacity of *G*. *truncatula* from high altitude endemic areas to survive up to more than 4 months after the end of the shedding period (Table 2) should be highlighted [48].

The cercarial shedding pattern detected in the Uruguayan couple does not disagree with the patterns observed by other authors on the *F*. *hepatica*/*G*. *truncatula* model [124,134–136]. When considering the shorter shedding period in the Uruguayan couple, the three shedding acrophases it shows (Fig 7A and 7C) fit well in the 4–5 shedding waves showed by the majority of *G*. *truncatula* in an experiment under constant conditions [124]. The delayed acrophases in *F*. *hepatica*/*L*. *neotropica* from Uruguay also agree with the pattern found in the European model. Here again, the pronounced differences of the couple from the Uruguayan lowlands with the *F*. *hepatica*/*G*. *truncatula* couple from the highlands of Bolivia [48] should be highlighted: regarding (i) the daily number of cercariae/snail, (ii) length of the shedding period, (iii) daily number of cercariae/snail, and (iv) cercarial production by the different redial generations. The longer post-infection longevity of *G*. *truncatula* under high altitude conditions [48,49] and the higher pathogenicity induced by the fast development and massive infection by *F*. *hepatica* in *L*. *neotropica* from Uruguayan lowlands underlie the aforementioned differences.

Summing up, our experimental results demonstrate that the *F*. *hepatica*/*L*. *neotropica* couple from Uruguayan lowlands is markedly less efficient for the disease transmission than the *F*. *hepatica*/*G*. *truncatula* couple from the Andean highlands, although somewhat more efficient than the *F*. *hepatica*/*G*. *truncatula* couple from European lowlands. The latter result agrees with other experimental data indicating that *L*. *neotropica* and *L*. *viator* from Argentina are better hosts than European (French) *G*. *truncatula* in both allopatric and sympatric infections by Argentinian and French isolates of *F*. *hepatica* [67]. The efficiency results of both, our present study of the Uruguayan couple and the one of the Argentinian and French mixed couples [67], may be interpreted taking into account that allopatric combinations of *F*. *hepatica* and lymnaeid species were proved to be more efficient than sympatric ones [137]. This capacity may be considered a useful strategy of the liver fluke for the colonization of new areas [6]. Indeed, the aforementioned historical analysis suggests that *L*. *neotropica* did not colonize Uruguay until at a maximum the first part of the 17th century, and consequently, around only 400 years ago. This is a very short period for the parasite from the evolutionary point of view. The aforementioned (i) Manter's rule [80], (ii) similarity of ancestral European livestock and present Uruguayan hosts, and (iii) low evolutionary rates of DNA markers used [81], should again be considered in this assumption. So, the term "allopatric" should be applied with caution here.

### Human fascioliasis reports

Only a total of 95 human fascioliasis cases have been reported in Uruguay. The first report of a human subject infected by *F*. *hepatica* in Uruguay was in 1909 and concerned a 49-year-old women suffering from right hypocondrium pain and in whom a fluke was unexpectedly found near the main biliary duct during surgery [138]. Twenty years later, three liver fluke specimens were detected during a gall bladder surgical intervention of another patient [139]. Between 1935 and 1950, isolated human cases were reported after egg detection in stool samples and/or duodenal exploration [140–142].

A familiar outbreak involving 11 members, probably linked to contaminated watercress consumption, was one year later diagnosed in the hospital of Paysandu. An exhaustive clinical and epidemiological study was performed [143].

The flooding events during the 1954/55 and 1958/59 periods were suggested to have spread fascioliasis and therefore related to three subsequently reported human epidemics [35]. Thus, a total of 31 cases were reported from the Florida department in 1960 [144], 20 additional cases were compiled from seven different departments from the country inland, the majority from Florida, Canelones and San José in 1978 [145], and finally another 16 cases diagnosed in an hospital during the 1953–1977 period were anatomo-pathologically described in 1979 [146].

Interestingly, among a review of patients infected by the HIV virus during the 1983–1988 period, *F*. *hepatica* eggs were found in the stools of a patient affected by AIDS and dying after 35 days hospitalization [147]. Only two additional cases were detected among a total of 951 samples during a wide serological and coprological survey performed in several localities of the departments of Artigas, Rivera, Florida and Salto throughout 1991 and 1993 (Lopez Lemes et al., 1992 and 1993, in [35]). Another infected patient with fever and eosinophilia was noted to be diagnosed both coprologically and immunologically in 1990 by E. Zanetta, in the same article [35].

Another familiar outbreak involving only three subjects and linked to the consumption of wild watercress, was reported (Lopez Lemes et al., 1994, in [35]). Finally, the last report was in 2003 about two female and one male clinical cases presenting with right hypocondrium pain, eosinophilia and history of watercress consumption [148].

### Human fascioliasis infection risk

The aforementioned review suggest a sporadic and isolated human infection risk throughout a wide hyperendemic animal fascioliasis zone in Uruguay, according to the epidemiological classification of WHO [149]. Several aspects merit, however, to be considered.

Despite the distribution of the liver fluke covering the whole country [37], the human infection risk does not appear to be homogeneous, i.e. it seems to be higher in given departments, as in Florida department [144,145]. Moreover, the unexpected finding of human infection in surgical interventions [138,139], HIV-infected patient survey [147], and wide surveys [35], suggest underestimation of the real occurrence of human infection, similarly as in Argentina [150]. The familiar outbreaks also remember the human fascioliasis situation in the lowlands of Argentina. However, whereas in the physiographically highly heterogeneous Argentina available data suggested human endemic local areas which have been finally described [51], the physiographic uniformity of Uruguay does not indicate such a scenario.

Nevertheless, the three increases of patient numbers after the flooding events of the 1954/55 and 1958/59 periods [144–146] should be considered by the public health responsibles, given IPCC (Intergovernmental Panel on Climate Change) predictions of a rainfall increase within the future climate change impact affecting Uruguay [68]. Anyway, there is nothing indicating a trend towards a worrying human infection scenario such as in Andean areas. Neither the ecological characteristics and preferences of the main vector *L*. *neotropica* [64], nor its transmission capacity verified in the present study, suggest such a future possibility. This does not mean, however, that the very fast larval development of *F*. *hepatica* and short shedding of high numbers of cercariae furnished by *L*. *neotropica* may take advantage of occasional, more or less prolonged flooding events to increase offspring, population densities and subsequently spread, thus enabling for an increase of familiar outbreaks or short transient epidemic situations.

The comparison of the transmission characteristics and capacities in the *F*. *hepatica*/*G*. *truncatula* couple from Bolivia with the *F*. *hepatica*/*L*. *neotropica* couple from Uruguay allow for the understanding of the high transmission patterns and endemicity characteristics of human fascioliasis in Andean highlands, opposite to the rare/sporadic/low human infection in animal endemic areas in Uruguayan lowlands. Consequently, it may be concluded that *L*. *neotropica* may be responsible for a human endemic area only under special circumstances, as in isolated foci in aridity/dryness areas described in Argentina [51].

The transmission characteristics and capacities of the Uruguayan *F*. *hepatica*/*L*. *neotropica* couple are a priori better for a seasonal transmission of the disease, depending on local climatic features. Uruguay has a subtropical to temperate climate with very marked seasonal fluctuations [69]. The climate is sub-humid, because potential evapotranspiration in summer is greater than precipitation. Although rainfall is distributed throughout the year, great variations occur between years. The highest precipitation occurs, in general, in summer and autumn. In the first season, precipitation is very irregular, with summers lacking precipitation and others with more than 600 mm of rain. In the second season, precipitation shows minor variability. Although precipitation has a somewhat smaller volume in winter than in other seasons, there is no marked rainy season. The great rainfall variation, both in regularity and intensity, should be highlighted because it leads to droughts and floods in different seasons of the year. Mean temperatures of the coldest month (July) are 10.8°C and 13.0°C, and the warmest month averages (January) are 22.6°C and 25.1°C for the Southern and Northern regions, respectively [69].

In Uruguay, field studies indicated that the fluke life cycle is maintained throughout the whole year, although it considerably slows down in winter [35]. Lymnaeids naturally infected in autumn-winter, with mean maximum temperatures lower than 20°C and mean minimum ones below 10°C, showed a 4–8 month cercarial shedding, whereas this was reduced to only 37 days in summer [63]. In spring, shedding periods gradually shorten, which together with an increase of lymnaeid population densities at the end of spring, gives rise to an increase of the number of infected animals at the end of spring and summer [38]. In summer, temperatures are ideal for *F*. *hepatica* development, but the insufficient rainfall and high evapotranspiration resulting in a humidity shortage become important limiting factors for the lymnaeids [40]. The long survival and infectivity of metacercariae [151] also add to understand the human infection risk the year long, despite it being higher in spring.
